# Integrin Trafficking and Tumor Progression

**DOI:** 10.1155/2012/516789

**Published:** 2011-10-30

**Authors:** Sejeong Shin, Laura Wolgamott, Sang-Oh Yoon

**Affiliations:** Department of Cancer and Cell Biology, College of Medicine, University of Cincinnati, Cincinnati, OH 45267, USA

## Abstract

Integrins are major mediators of cancer cell adhesion to extracellular matrix. Through this interaction, integrins play critical roles in cell migration, invasion, metastasis, and resistance to apoptosis during tumor progression. Recent studies highlight the importance of integrin trafficking, endocytosis and recycling, for the functions of integrins in cancer cells. Understanding the molecular mechanisms of integrin trafficking is pivotal for understanding tumor progression and for the development of anticancer drugs.

## 1. Integrins and Cancer

Most of the cells in multicellular organisms are surrounded by a complex mixture of nonliving materials that make up the extracellular matrix (ECM). The ECM of vertebrates is composed of complex mixtures of proteins (collagens, laminins, fibronectin, etc.) and proteoglycans (chondroitin sulfate, keratan sulfate, etc.) [1, 2]. ECM plays a significant role in regulating numerous cellular functions, including cell shape, adhesion, migration, proliferation, polarity, differentiation, and apoptosis [1]. In pathological conditions such as cancer, increased synthesis of certain ECM components and/or increased breakdown with consequent generation of ECM cleavage products can contribute to cancer growth and progression [3]. 

Cells attach to the ECM by means of integrins. Integrins are transmembrane glycoproteins that are composed of a set of noncovalently associated *α* and *β* subunits. There are at least 18*α* and 8*β* subunits capable of forming more than 24*αβ* heterodimers that account for the structural and functional diversity of the integrin family [4–6]. Integrins consist of a large extracellular domain, a single transmembrane domain, and a cytoplasmic tail [7]. The extracellular portion of integrins binds to ECM proteins, and the intracellular portion binds to cellular cytoskeletal elements such as actin filaments. This connection reinforces tissue integrity and cell adhesion and stabilizes cell protrusions during migration. The connection also constitutes a signaling platform through which integrins can relay information for major processes such as transcriptional control, cell death, proliferation, and cell migration [8, 9]. There is a growing body of evidence suggesting that alterations in the adhesion properties of neoplastic cells endow them with an invasive and migratory phenotype. Indeed, changes in the expression and/or function of integrins have been implicated in all steps of tumor progression, including detachment of tumor cells from the primary site, invasion of ECM, intravasation into the blood stream, dissemination through the circulation, extravasation into distant target organs, and formation of the secondary lesions [5, 10–13]. 

Although at least 24*αβ* integrin heterodimers are known, *α*5*β*1, *α*6*β*4, *α*v*β*3, and *α*v*β*6 integrins have been extensively studied in cancer and their expression is correlated with cancer progression in various tumor types [14–18]. Upregulation of these integrins renders cancer cells more motile, invasive, and resistant to anticancer drugs [5, 18]. Unlike these integrins, expression levels of some integrins, such as *α*2*β*1 and *α*1*β*1, decrease in tumor cells, which potentially increase tumor cell dissemination [18–21]. In addition to changes in expression, changes in the function of these integrins also play a critical role in cancer progression.

## 2. Integrin Trafficking

The way in which integrins are trafficked by the endosomal pathway is now recognized to influence their function [6, 22]. Certain integrin heterodimers are continually internalized from the plasma membrane into endosomal compartments and subsequently recycled back to the cell surface, which indicates that the endocytic and recycling pathways have the potential to exert minute-to-minute control over integrin function. Abundant evidence suggests that integrin trafficking regulates cell adhesion to ECM, establishes and maintains cell polarity, redefines signaling pathways, and controls migration [6, 23]. Therefore, transcriptional changes, mutational alterations, and deregulated cellular signaling changing endocytosis and recycling of integrins confer invasive and metastatic properties to tumor cells. 

**Figure 1:**
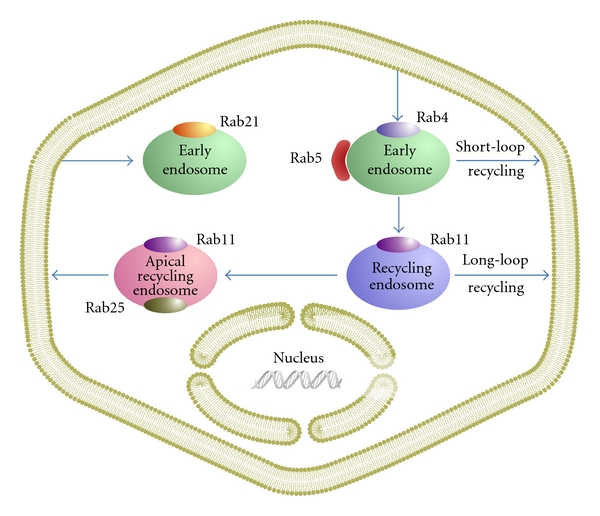
The roles of Rab GTPases involved in integrin trafficking. Integrins can be internalized by a clathrin-dependent, caveolin-dependent, or clathrin- and caveolin-independent pathway. For example, some integrins are internalized by a Rab21 and clathrin-independent pathway. Once internalized, integrins can be recycled back to the plasma membrane by a Rab4-dependent manner or can be transported to the perinuclear recycling compartment. Rab11 family members (Rab11 and Rab25) regulate recycling of integrins from perinuclear recycling compartment.

Integrin trafficking is regulated by members of the Ras-associated binding (Rab) family of small GTPases, which function as molecular switches regulating vesicular transport in eukaryotic cells [24, 25] (Figure 1). Through their indirect interactions with coat components, motors, and other proteins, the Rab GTPases serve as multifaceted organizers of almost all membrane trafficking processes including integrin trafficking [25, 26]. Approximately 70 types of Rab GTPases have now been identified in humans [27]. Among these, several Rab GTPases regulate endocytosis and recycling of integrins. For example, Rab21 mediates integrin endocytosis (Figure 1). In addition, Rab11 mediates slow integrin recycling through recycling endosomes, whereas Rab4 mediates fast integrin recycling directly from early endosomes (Figure 1) [25]. 

The pathophysiological roles of Rab GTPases in human malignancies have been less studied compared to members of the Ras and Rho GTPase families. However, more attention has been paid to the roles of Rab GTPases in cancer in recent years, and several members of the Rab family such as Rab11 and Rab25 have been shown to be aberrantly expressed in various cancer types [25, 28, 29]. Because of the important roles of Rab GTPases in integrin trafficking, deregulation of Rab GTPases is closely related to cancer development and progression [24, 25, 29].

### 2.1. Integrin Endocytosis

There are several major endocytosis mechanisms, including clathrin-mediated endocytosis, caveolae-mediated endocytosis, and clathrin- and caveolin-independent endocytosis [30–33]. Clathrin-mediated endocytosis is mediated by small vesicles that have a morphologically characteristic crystalline coat made up of a complex of proteins associated with the cytosolic protein clathrin [33]. Clathrin-coated vesicles (CCVs) are found in virtually all cells and form domains of the plasma membrane termed clathrin-coated pits. Clathrin-coated pits can concentrate large extracellular molecules and receptors responsible for the receptor-mediated endocytosis of ligands, for example, low-density lipoprotein, transferrin, growth factors, and antibodies [30, 33]. In contrast, caveolae-mediated endocytosis is mediated by small flask-shape pits, caveolae, in the membrane. Caveolae are the most common reported non-clathrin-coated plasma membrane buds which exist on the surface of many, but not all cell types [33]. They consist of the cholesterol-binding protein caveolin (Vip21) with a bilayer enriched in cholesterol and glycolipids [30]. Clathrin- and caveolin-independent endocytosis includes macropinocytosis and circular dorsal ruffles [9, 30, 33]. Clathrin-dependent endocytosis and caveolin-dependent endocytosis require dynamin and exhibit small vesicles. However, macropinocytosis does not require dynamin and displays highly ruffled structures. Like macropinocytosis, circular dorsal ruffles show highly ruffled structures, but this endocytosis is dependent on dynamin. 

Integrins are known to be endocytosed by clathrin-mediated endocytosis, caveolae-mediated endocytosis, and clathrin- and caveolin-independent endocytosis (Table 1). It is probable that a given type of integrin heterodimer follows more than one route to internalization depending on regions within a cell, cell conditions, and cell type [6, 34]. For instance, a subpopulation of integrin *α*5*β*1 is internalized into clathrin-coated structures near focal complexes at the cell front, whereas the bulk of integrin *α*5*β*1 follows a nonclathrin pathway from other parts of the cell surface [34].

Deregulation of integrin endocytosis is closely related to cancer development and progression [9, 30]. For example, chromosomal deletion and loss of Rab21, a regulator of endocytic trafficking of integrins, has been found in cancer that leads to the accumulation of multinucleate cells in cancer. The correlation with multinucleate cells is thought to reflect the requirement of Rab21-mediated integrin endocytosis for correct cytokinesis [35]. Rab21 also enhances cancer cell adhesion and migration by regulating integrin endocytosis [36].

### 2.2. Integrin Recycling

Once internalized, integrins are predominantly recycled back to the plasma membrane, although a fraction of integrin *α*5*β*1 has been shown to traffic to lysosomes for degradation during migration [6, 37]. Following endocytosis, integrins travel to early endosomes from which they can either be returned directly to the plasma membrane in a Rab4-dependent manner (the short loop) or further trafficked to the perinuclear recycling compartment (PNRC) before recycling through Rab11-dependent mechanisms (the long loop) (Table 1). Rab11 GTPase functions have been linked to tumorigenesis and tumor progression. Rab11 is upregulated during skin carcinogenesis [38] and is linked to Barrett's dysplasia [39]. However, the function of the Rab11 family member Rab25 (or Rab11C) is controversial. Rab25 shows highly restricted expression under normal physiological conditions but is upregulated in invasive cancer cell lines and metastatic tumor cells [40], and its elevated expression is further linked to the aggressiveness of breast and ovarian cancers [28]. Rab25 is a determinant of tumor progression, and the aggressiveness of epithelial cancers and is strongly associated with decreased survival [28]. In contrast, recent studies showed that Rab25 expression is decreased in human colon cancers and triple-negative (negative for estrogen receptor (ER), progesterone receptor (PR), and Her2/Neu) breast cancers, and Rab25 functions as a tumor suppressor in these cancers [41–43]. The key roles of Rab GTPases in tumorigenesis and tumor progression are closely related to integrin recycling. For example, Rab25 contributes to tumor progression by directing the localization of integrin-recycling vesicles and thereby enhancing the ability of tumor cells to invade the extracellular matrix [44].

## 3. Trafficking of Integrin ***α***5***β***1

Integrin *α*5*β*1 is a receptor for fibronectin and contributes to cancer cell invasion, metastasis, resistance to anticancer drugs, and decreased survival in patients [17, 45]. 

Integrin *α*5*β*1 is internalized by clathrin-dependent, caveolin-dependent, and clathrin- and caveolin-independent mechanisms. For clathrin-dependent endocytosis, *α*5*β*1 integrin binds to NUMB, an endocytic protein that influences clathrin-coated pit assembly [46]. Integrin *α*5*β*1 can also internalize with tetraspanin protein, which interacts with AP-2, an adaptor for clathrin-mediated endocytosis [47]. Clathrin-dependent internalization of *α*5*β*1 integrin with NUMB or tetraspanin has a profound effect on cell migration. In addition, *α*5*β*1 integrin can undergo Rab21- and clathrin-independent endocytosis that is required for successful cytokinesis [35]. In some cell types, integrin *α*5*β*1 localizes to caveolae for caveolin-mediated endocytosis [6, 48]. Caveolin-dependent endocytosis of integrin *α*5*β*1 is critical for fibronectin turnover [48].

Internalized integrin *α*5*β*1 is transported through Rab4-positive early endosomes and arrives at the Rab11-positive perinuclear recycling compartment [49]. Akt-mediated glycogen synthase kinase (GSK)-3 phosphorylation is known to deliver *α*5*β*1 from the Rab11 compartment to the plasma membrane [50]. One of the Rab11 effectors, Rab11 FIP1/RCP, associates with integrin *α*5*β*1 and regulates recycling of this integrin [51]. Rab-coupling protein (RCP) provides a scaffold that promotes the physical association and coordinated trafficking of *α*5*β*1 and epidermal growth factor receptor 1 (EGFR1). This association drives migration of tumor cells into three-dimensional matrices [51]. Recently, it was shown that mutant p53 can promote invasion, loss of directionality of migration, and metastatic behavior by regulating the interaction of *α*5*β*1 integrin to Rab-coupling protein, which enhances *α*5*β*1 trafficking and signaling [52]. Since Rab25 (Rab11C, Rab11 family member) binds to Rab11 FIP1/RCP, it is hypothesized that interaction between them may control integrin *α*5*β*1 trafficking. Recently, it has been shown that Rab25 associates with *α*5*β*1 integrin to enhance migration and invasion of cells in three-dimensional microenvironments and directs *α*5*β*1 integrin recycling to dynamic ruffling protrusions at the leading edge of migrating cells, which promotes invasive migration [29, 44]. In addition to Rab11 and Rab25, Rab21 is required for carcinoma-associated fibroblasts to promote invasion by cancer cells and facilitates integrin *α*5*β*1 accumulation for force-mediated matrix remodeling at the plasma membrane [53]. It has also been shown that Rab21-dependent recycling of integrin *α*5*β*1 is critical for proper activation of RhoA during cytokinesis [35]. 

Although most endocytosed integrin *α*5*β*1 is known to recycle back to plasma membrane, a subset of this integrin moves to lysosomes for degradation [37]. This process is very slow, but it is important for *α*5*β*1-dependent cell motility [37].

## 4. Trafficking of Integrin ***α***6***β***4

Integrin *α*6*β*4 is a receptor for laminin. Overexpression of *α*6*β*4 integrin was seen in several types of cancers including breast cancer and correlated with tumor invasion, increased tumor size and grade, and a poor prognosis [54–57]. 

We showed that integrin *α*6*β*4 integrin recycles back to the plasma membrane via the Rab11-positive perinuclear recycling compartment [58]. Hypoxia stimulated carcinoma invasion by promoting Rab11 trafficking of integrin *α*6*β*4, which is dependent on hypoxia-inhibited glycogen synthase kinase (GSK)-3 signaling [58].

## 5. Trafficking of Integrin ***α***v***β***3

Integrin *α*v*β*3 is a receptor for fibronectin and vitronectin. Integrin *α*v*β*3 is expressed in a variety of tumors such as melanoma, prostate cancer, and breast cancer [59]. Integrin *α*v*β*3 is overexpressed in activated endothelial cells during tumor-induced angiogenesis, whereas it is absent on quiescent endothelial cells and normal tissues. It is known that integrin *α*v*β*3 promotes cancer cell survival, migration, invasion, and metastasis [4, 60, 61]. 

Integrin *α*v*β*3 is endocytosed via clathrin-dependent, caveolin-dependent, or clathrin- and caveolin-independent mechanisms. NUMB is an alternative clathrin adaptor and can interact with *β*3 integrin, which controls *α*v*β*3 integrin endocytosis and cell migration [46]. In some cell types, integrin *α*v*β*3 is internalized by caveolin-dependent mechanisms [62]. In this case, MT1-MMP is clustered together with caveolin-1 and *α*v*β*3 integrin at motility-associated structures, resulting in increased proteolytic activity, which is important for cell migration and invasion [62]. A recent study shows that upon growth factors stimulation, integrin *β*3 abruptly redistributes to circular dorsal ruffles where it is internalized through macropinocytosis, which plays an important role in growth factor-induced cell migration [63].

Internalized integrin *α*v*β*3 recycles back to the plasma membrane via Rab4-dependent mechanisms or the Rab11-positive perinuclear recycling compartment [23]. Following treatment with PDGF, integrin *α*v*β*3 was rapidly recycled directly back to the plasma membrane from early endosomes via a Rab4-dependent mechanism without the involvement of Rab11 [49]. The PKC-related kinase PKD1 influences cell migration by this fast recycling of integrin *α*v*β*3. It is known that PKD1 directly interacts with *β*3 integrin and this interaction promotes fast recycling of *α*v*β*3 integrin from recycling endosomes to the plasma membrane upon growth factor stimulation [64]. Activation of VEGFR1 also enhances a Rab4A-dependent pathway that transports *α*v*β*3-integrin from early endosomes to the plasma membrane [65]. Recent studies link PKD and VEGF signaling in which VEGF-A induces recycling of integrin *α*v*β*3 in a PKD1-dependent manner [66]. Because of the involvement of Rab4 in the recycling of *α*v*β*3 integrin, inhibition of Rab4 effector protein (Rab IP4) blocks integrin recycling, leading to inhibition of cell adhesion and cell spreading [67]. Another study suggests that supervillin, an actin and myosin binding protein, regulates rapid *β*3 integrin recycling through collaboration with Rab4 and Rab5 [68]. The short-loop recycling of integrin *α*v*β*3 via Rab4 does not directly contribute to migration by moving *α*v*β*3 to the cell front, but by antagonizing *α*5*β*1 recycling, which, in turn, influences the cell's decision to migrate with persistence or to move randomly [69]. Integrin *α*v*β*3 is also recycled to the plasma membrane in a Rab11-dependent manner (long loop recycling) in which Akt promotes this recycling by phosphorylating and inactivating GSK-3 [50].

## 6. Trafficking of Integrin ***α***v***β***6

Integrin *α*v*β*6 is a receptor for fibronectin, vitronectin, and tenascin. Integrin *α*v*β*6 is usually expressed at low or undetectable levels in most healthy adult epithelia but is upregulated in many cancers such as colon cancer [70]. The expression of integrin *α*v*β*6 inhibits apoptosis and promotes tumor cell invasion and metastasis, which is often associated with a more aggressive disease outcome and a poor prognosis [18, 70]. 

Recently, the mechanism of endocytosis of integrin *α*v*β*6 was revealed. Integrin *α*v*β*6 is internalized by a clathrin-dependent mechanism by interaction with HS1-associated protein X1 (HAX1) [71]. HAX1 is found in clathrin-coated vesicles, and the cytodomain of *β*6 integrin interacts with HAX1 and is endocytosed, which increases carcinoma migration and invasion [71].

## 7. Conclusion and Future Direction

Integrins are key regulators of cell adhesion, migration, and proliferation. Therefore, deregulation of their expression and altered functions play critical roles in cancer progression by enhancing cancer cell invasion, metastasis, and survival. There are now clear indications that integrin trafficking is important to modulate integrin distribution and function. However, more studies are needed to define the molecular mechanisms of integrin trafficking in tumor progression. Many questions remain to be answered. One important question is whether endosomal integrins can signal cell proliferation and migration. It is known that unligated integrins can positively or negatively regulate tumor cell survival and metastasis, and, therefore, signaling arising from endosomal compartments may be important for tumor cell survival. Another question is how trafficking of specific integrins affects other integrins. For instance, it has been shown that rapid recycling of *α*v*β*3 via the Rab4 pathway antagonizes the Rab11-mediated *α*5*β*1 recycling, which influences the cell's decision to migrate with persistence or to move randomly [69]. Because of the critical roles of integrins in cancer progression, integrins are potential targets for the development of targeted anticancer therapeutics. Understanding the mechanism of integrin trafficking will provide valuable information for the development of new anticancer drugs and clues to increase the efficacy of current anticancer therapeutics.

## Figures and Tables

**Table 1:** Mechanisms of the internalization and recycling of integrins *α*5*β*1, *α*6*β*4, *α*v*β*3, and *α*v*β*6.

Integrin	Internalization		Recycling	
	Associated proteins	Comments	Associated proteins	comments
*α*5*β*1	Numb	Clathrin-dependent	Rab11	Akt/GSK-3*β*-dependent
	AP2 associated with tetraspanin	Clathrin-dependent	Rab11 RCP	Akt-dependent
	NRP1	Clathrin-dependent	VAMP3	SNARE-mediated
	Rab21	Clathrin-dependent	Rab21	Required for cytokinesis
	?	Caveolin-dependent	Rab25	Promote invasion in 3D

*α*6*β*4			Rab11	Akt/GSK-3*β*-dependent

*α*v*β*3	Numb ?	Clathrin-dependent Caveolin-dependent	Rab4, PKD1 Rab4, RABIP4 Rab11	PDGF- or VEGF-driven PDGF-driven Akt/GSK-3-dependent

*α*v*β*6	HAX1	Clathrin-dependent		

## References

[1] Jean C, Gravelle P, Fournie JJ, Laurent G (2011). Influence of stress on extracellular matrix and integrin biology. *Oncogene*.

[2] Dufort CC, Paszek MJ, Weaver VM (2011). Balancing forces: architectural control of mechanotransduction. *Nature Reviews Molecular Cell Biology*.

[3] Pupa SM, Ménard S, Forti S, Tagliabue E (2002). New insights into the role of extracellular matrix during tumor onset and progression. *Journal of Cellular Physiology*.

[4] Cox D, Brennan M, Moran N (2010). Integrins as therapeutic targets: lessons and opportunities. *Nature Reviews Drug Discovery*.

[5] Makrilia N, Kollias A, Manolopoulos L, Syrigos K (2009). Cell adhesion molecules: role and clinical significance in cancer. *Cancer Investigation*.

[6] Caswell PT, Vadrevu S, Norman JC (2009). Integrins: masters and slaves of endocytic transport. *Nature Reviews Molecular Cell Biology*.

[7] Luo BH, Carman CV, Springer TA (2007). Structural basis of integrin regulation and signaling. *Annual Review of Immunology*.

[8] Ulrich F, Heisenberg CP (2009). Trafficking and cell migration. *Traffic*.

[9] Ramsay AG, Marshall JF, Hart IR (2007). Integrin trafficking and its role in cancer metastasis. *Cancer and Metastasis Reviews*.

[10] Felding-Habermann B, O’Toole TE, Smith JW (2001). Integrin activation controls metastasis in human breast cancer. *Proceedings of the National Academy of Sciences of the United States of America*.

[11] Bates RC, Bellovin DI, Brown C (2005). Transcriptional activation of integrin *β*6 during the epithelial-mesenchymal transition defines a novel prognostic indicator of aggressive colon carcinoma. *Journal of Clinical Investigation*.

[12] Mercurio AM, Rabinovitz I, Shaw LM (2001). The *α*6*β*4 integrin and epithelial cell migration. *Current Opinion in Cell Biology*.

[13] Brooks PC, Strömblad S, Sanders LC (1996). Localization of matrix metalloproteinase MMP-2 to the surface of invasive cells by interaction with integrin *α*v*β*3. *Cell*.

[14] Nip J, Shibata H, Loskutoff DJ, Cheresh DA, Brodt P (1992). Human melanoma cells derived from lymphatic metastases use integrin *α*(v)*β*3 to adhere to lymph node vitronectin. *Journal of Clinical Investigation*.

[15] Friedrichs K, Ruiz P, Franke F, Gille I, Terpe HJ, Imhof BA (1995). High expression level of *α*6 integrin in human breast carcinoma is correlated with reduced survival. *Cancer Research*.

[16] McCabe NP, De S, Vasanji A, Brainard J, Byzova TV (2007). Prostate cancer specific integrin *α*v*β*3 modulates bone metastatic growth and tissue remodeling. *Oncogene*.

[17] Adachi M, Taki T, Higashiyama M, Kohno N, Inufusa H, Miyake M (2000). Significance of integrin *α*5 gene expression as a prognostic factor in node-negative non-small cell lung cancer. *Clinical Cancer Research*.

[18] Desgrosellier JS, Cheresh DA (2010). Integrins in cancer: biological implications and therapeutic opportunities. *Nature Reviews Cancer*.

[19] Kren A, Baeriswyl V, Lehembre F (2007). Increased tumor cell dissemination and cellular senescence in the absence of *β*1-integrin function. *EMBO Journal*.

[20] Ramirez NE, Zhang Z, Madamanchi A (2011). The *α*2*β*1 integrin is a metastasis suppressor in mouse models and human cancer. *Journal of Clinical Investigation*.

[21] Matilla E, Pellinen T, Nevo J, Vuoriluoto K, Arjonen A, Ivaska J (2005). Negative regulation of EGFR signalling through integrin-*α*1*β*1-mediated activation of protein tyrosine phosphatase TCPTP. *Nature Cell Biology*.

[22] Pellinen T, Ivaska J (2006). Integrin traffic. *Journal of Cell Science*.

[23] Caswell PT, Norman JC (2006). Integrin trafficking and the control of cell migration. *Traffic*.

[24] Chia WJ, Tang BL (2009). Emerging roles for Rab family GTPases in human cancer. *Biochimica et Biophysica Acta—Reviews on Cancer*.

[25] Stenmark H (2009). Rab GTPases as coordinators of vesicle traffic. *Nature Reviews Molecular Cell Biology*.

[26] Jones MC, Caswell PT, Norman JC (2006). Endocytic recycling pathways: emerging regulators of cell migration. *Current Opinion in Cell Biology*.

[27] Mitra S, Cheng KW, Mills GB (2011). Rab GTPases implicated in inherited and acquired disorders. *Seminars in Cell and Developmental Biology*.

[28] Cheng KW, Lahad JP, Kuo WL (2004). The RAB25 small GTPase determines aggressiveness of ovarian and breast cancers. *Nature Medicine*.

[29] Subramani D, Alahari SK (2010). Integrin-mediated function of Rab GTPases in cancer progression. *Molecular Cancer*.

[30] Mosesson Y, Mills GB, Yarden Y (2008). Derailed endocytosis: an emerging feature of cancer. *Nature Reviews Cancer*.

[31] Sorkin A, von Zastrow M (2009). Endocytosis and signalling: intertwining molecular networks. *Nature Reviews Molecular Cell Biology*.

[32] Grant BD, Donaldson JG (2009). Pathways and mechanisms of endocytic recycling. *Nature Reviews Molecular Cell Biology*.

[33] Doherty GJ, McMahon HT (2009). Mechanisms of endocytosis. *Annual Review of Biochemistry*.

[34] Caswell P, Norman J (2008). Endocytic transport of integrins during cell migration and invasion. *Trends in Cell Biology*.

[35] Pellinen T, Tuomi S, Arjonen A (2008). Integrin trafficking regulated by Rab21 is necessary for cytokinesis. *Developmental Cell*.

[36] Pellinen T, Arjonen A, Vuoriluoto K, Kallio K, Fransen JAM, Ivaska J (2006). Small GTPase Rab21 regulates cell adhesion and controls endosomal traffic of *β*1-integrins. *Journal of Cell Biology*.

[37] Lobert VH, Brech A, Pedersen NM (2010). Ubiquitination of *α*5*β*1 integrin controls fibroblast migration through lysosomal degradation of fibronectin-integrin complexes. *Developmental Cell*.

[38] Gebhardt C, Breitenbach U, Richter KH (2005). c-Fos-dependent induction of the small ras-related GTPase Rab11a in skin carcinogenesis. *American Journal of Pathology*.

[39] Goldenring JR, Ray GS, Lee JR (1999). Rab11 in dysplasia of Barrett’s epithelia. *Yale Journal of Biology and Medicine*.

[40] Wang W, Goswami S, Lapidus K (2004). Identification and testing of a gene expression signature of invasive carcinoma cells within primary mammary tumors. *Cancer Research*.

[41] Cheng JM, Volk L, Janaki DKM, Vyakaranam S, Ran S, Rao KA (2010). Tumor suppressor function of Rab25 in triple-negative breast cancer. *International Journal of Cancer*.

[42] Goldenring JR, Nam KT (2011). Rab25 as a tumour suppressor in colon carcinogenesis. *British Journal of Cancer*.

[43] Nam KT, Lee HJ, Smith JJ (2010). Loss of Rab25 promotes the development of intestinal neoplasia in mice and is associated with human colorectal adenocarcinomas. *Journal of Clinical Investigation*.

[44] Caswell PT, Spence HJ, Parsons M (2007). Rab25 associates with *α*5*β*1 integrin to promote invasive migration in 3D microenvironments. *Developmental Cell*.

[45] Soung YH, Clifford JL, Chung J (2010). Crosstalk between integrin and receptor tyrosine kinase signaling in breast carcinoma progression. *BMB Reports*.

[46] Nishimura T, Kaibuchi K (2007). Numb controls integrin endocytosis for directional cell migration with aPKC and PAR-3. *Developmental Cell*.

[47] Liu L, He B, Liu WM, Zhou D, Cox JV, Zhang XA (2007). Tetraspanin CD151 promotes cell migration by regulating integrin trafficking. *Journal of Biological Chemistry*.

[48] Shi F, Sottile J (2008). Caveolin-1-dependent *β*1 integrin endocytosis is a critical regulator of fibronectin turnover. *Journal of Cell Science*.

[49] Roberts M, Barry S, Woods A, Van der Sluijs P, Norman J (2001). PDGF-regulated rab4-dependent recycling of *α*v*β*3 integrin from early endosomes is necessary for cell adhesion and spreading. *Current Biology*.

[50] Roberts MS, Woods AJ, Dale TC, van der Sluijs P, Norman JC (2004). Protein kinase B/Akt acts via glycogen synthase kinase 3 to regulate recycling of *α*v*β*3 and *α*5*β*1 integrins. *Molecular and Cellular Biology*.

[51] Caswell PT, Chan M, Lindsay AJ, McCaffrey MW, Boettiger D, Norman JC (2008). Rab-coupling protein coordinates recycling of *α*5*β*1 integrin and EGFR1 to promote cell migration in 3D microenvironments. *Journal of Cell Biology*.

[52] Muller PAJ, Caswell PT, Doyle B (2009). Mutant p53 drives invasion by promoting integrin recycling. *Cell*.

[53] Hooper S, Gaggioli C, Sahai E (2010). A chemical biology screen reveals a role for Rab21-mediated control of actomyosin contractility in fibroblast-driven cancer invasion. *British Journal of Cancer*.

[54] Guo W, Pylayeva Y, Pepe A (2006). *β*4 integrin amplifies ErbB2 signaling to promote mammary tumorigenesis. *Cell*.

[55] Weaver VM, Lelièvre S, Lakins JN (2002). *β*4 integrin-dependent formation of polarized three-dimensional architecture confers resistance to apoptosis in normal and malignant mammary epithelium. *Cancer Cell*.

[56] Mercurio AM, Rabinovitz I (2001). Towards a mechanistic understanding of tumor invasion—Lessons from the *α*6*β*4 integrin. *Seminars in Cancer Biology*.

[57] Diaz LK, Cristofanilli M, Zhou X (2005). *β*4 Integrin subunit gene expression correlates with tumor size and nuclear grade in early breast cancer. *Modern Pathology*.

[58] Yoon SO, Shin S, Mercurio AM (2005). Hypoxia stimulates carcinoma invasion by stabilizing microtubules and promoting the Rab11 trafficking of the *α*6*β*4 integrin. *Cancer Research*.

[59] Natali PG, Hamby CV, Felding-Habermann B (1997). Clinical significance of *α*(v)*β*3 integrin and intercellular adhesion molecule-1 expression in cutaneous malignant melanoma lesions. *Cancer Research*.

[60] Nemeth JA, Nakada MT, Trikha M (2007). Alpha-v integrins as therapeutic targets in oncology. *Cancer Investigation*.

[61] Koistinen P, Ahonen M, Kähäri VM, Heino J (2004). *α*V integrin promotes in vitro and in vivo survival of cells in metastatic melanoma. *International Journal of Cancer*.

[62] Gálvez BG, Matías-Román S, Yáñez-Mó M, Vicente-Manzanares M, Sánchez-Madrid F, Arroyo AG (2004). Caveolae are a novel pathway for membrane-type 1 matrix metalloproteinase traffic in human endothelial cells. *Molecular Biology of the Cell*.

[63] Gu Z, Noss EH, Hsu VW, Brenner MB (2011). Integrins traffic rapidly via circular dorsal ruffles and macropinocytosis during stimulated cell migration. *Journal of Cell Biology*.

[64] Woods AJ, White DP, Caswell PT, Norman JC (2004). PKD1/PKC*μ* promotes *α*v*β*3 integrin recycling and delivery to nascent focal adhesions. *EMBO Journal*.

[65] Jones MC, Caswell PT, Moran-Jones K (2009). VEGFR1 (Flt1) regulates Rab4 recycling to control fibronectin polymerization and endothelial vessel branching. *Traffic*.

[66] di Blasio L, Droetto S, Norman J, Bussolino F, Primo L (2010). Protein kinase D1 regulates VEGF-A-induced alphavbeta3 integrin trafficking and endothelial cell migration. *Traffic*.

[67] Vukmirica J, Monzo P, Le Marchand-Brustel Y, Cormont M (2006). The Rab4A effector protein Rabip4 is involved in migration of NIH 3T3 fibroblasts. *Journal of Biological Chemistry*.

[68] Fang Z, Takizawa N, Wilson KA (2010). The membrane-associated protein, supervillin, accelerates F-actin-dependent rapid integrin recycling and cell motility. *Traffic*.

[69] White DP, Caswell PT, Norman JC (2007). *α*v*β*3 and *α*5*β*1 integrin recycling pathways dictate downstream Rho kinase signaling to regulate persistent cell migration. *Journal of Cell Biology*.

[70] Bandyopadhyay A, Raghavan S (2009). Defining the role of integrin *α*v*β*6 in cancer. *Current Drug Targets*.

[71] Ramsay AG, Keppler MD, Jazayeri M (2007). HS1-associated protein X-1 regulates carcinoma cell migration and invasion via clathrin-mediated endocytosis of integrin *α*v*β* 6. *Cancer Research*.

